# Analysis of dislocation defects in compositionally step-graded α-(Al_*x*_Ga_1−*x*_)_2_O_3_ layers

**DOI:** 10.1039/d4ra06182a

**Published:** 2024-10-04

**Authors:** Tatsuya Yasuoka, Hiromu Susami, Li Liu, Giang T. Dang, Toshiyuki Kawaharamura

**Affiliations:** a School of Systems Engineering, Kochi University of Technology 185 Miyanokuchi, Tosayamada, Kami Kochi 782-8502 Japan; b Center for Nanotechnology, Research Institute, Kochi University of Technology 185 Miyanokuchi, Tosayamada, Kami Kochi 782-8502 Japan kawaharamura.toshiyuki@kochi-tech.ac.jp

## Abstract

The ultra-wide bandgap semiconductor α-Ga_2_O_3_ can be heteroepitaxially grown on a sapphire substrate. However, due to a lattice mismatch of about 4.6% with a sapphire substrate, many dislocation defects occur in α-Ga_2_O_3_ films. To reduce the dislocation density, compositionally step-graded α-(Al_*x*_Ga_1−*x*_)_2_O_3_ layers were fabricated on a *c*-plane sapphire substrate using mist CVD. TEM measurements revealed few dislocations in the initial layer of α-(Al_0.96_Ga_0.04_)_2_O_3_, but numerous dislocations were observed in the subsequent layer of α-(Al_0.84_Ga_0.16_)_2_O_3_. However, the step-graded α-(Al_*x*_Ga_1−*x*_)_2_O_3_ layers exhibited bending of the dislocations under both compressive and tensile strains due to compositional differences of α-(Al_*x*_Ga_1−*x*_)_2_O_3_, resulting in about 50% reduction of the dislocation density in the high-Ga-composition layer of α-(Al_*x*_Ga_1−*x*_)_2_O_3_. The introduction of multiple 50 nm α-Ga_2_O_3_ layers into the compositionally step-graded α-(Al_*x*_Ga_1−*x*_)_2_O_3_ layers resulted in a notable reduction in dislocation defects at the interface between the sandwiched α-Ga_2_O_3_ layers. It is assumed that the dislocations were bent by the strain caused by the composition change, resulting in a decrease in the number of dislocations. It is anticipated that further reduction of dislocation density will be achieved by optimizing the composition change and thicknesses of layers that provide effective strain for dislocation bending, and by stacking these layers.

## Abstract 2

The dislocations in α-(Al_*x*_Ga_1−*x*_)_2_O_3_ layers were bent by the strain caused by the composition change, resulting in a decrease in the number of dislocations.

## Graphical abstract

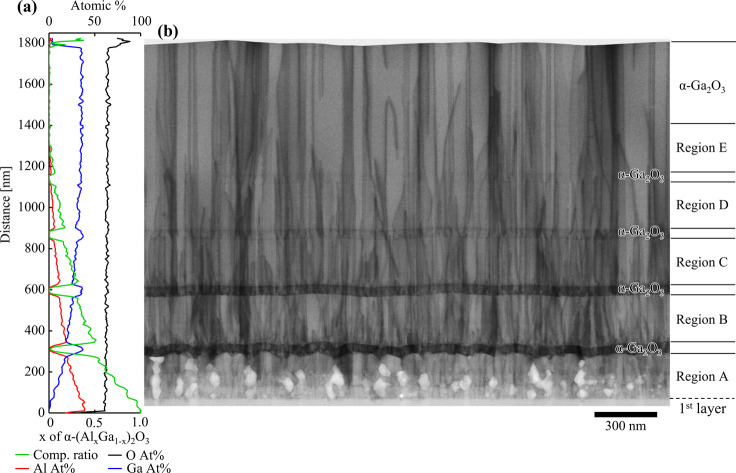

## 1. Introduction

Gallium oxide (Ga_2_O_3_) is gaining attention as an ultra-wide bandgap semiconductor material. Ga_2_O_3_ has various crystal structures,^1^ and among them, the thermally most stable phase is β-Ga_2_O_3_ with a band gap of ∼4.9 eV,^2,3^ which can be grown from melt.^4–7^ Thus, homoepitaxial growth is possible,^8–10^ and β-Ga_2_O_3_ has been the most actively studied. On the other hand, α-Ga_2_O_3_, which has a corundum structure and is thermally metastable, has the largest band gap of 5.3 eV (ref. 11) (5.61 eV),^12^ among Ga_2_O_3_ polymorphs of different crystal structures and can be heteroepitaxially grown on a sapphire substrate.^11,13–18^ It can be mixed with other corundum-structured oxides α-(M_*x*_Ga_1−*x*_)_2_O_3_ [M = Al,^19–24^ In,^25^ Fe,^26^ Cr,^27^ and Ir^28^] to control various properties such as band gaps and lattice constants, and is expected to be used for power devices. However, the sapphire substrate and α-Ga_2_O_3_ have about 4.60% lattice mismatch along the *a*-axis,^29^ and many dislocations are formed due to this large lattice mismatch.^30,31^ This high dislocation density is one of the most significant problems to be solved in α-Ga_2_O_3_ device applications. Previous studies have examined the Epitaxial Lateral Overgrowth (ELO) method^32–35^ and the introduction of α-(Al_*x*_Ga_1−*x*_)_2_O_3_ buffer layers^20,36,37^ to decrease dislocation density. In this study, we have attempted to fabricate compositionally step-graded α-(Al_*x*_Ga_1−*x*_)_2_O_3_ layers to reduce the dislocation density. Additionally, in a previous study, the dislocation density was reduced with the introduction of quasi-graded α-(Al_*x*_Ga_1−*x*_)_2_O_3_ buffer layers, although the mechanism behind it was unclear.^36^ This study revealed the mechanism by analyzing dislocations in the step-graded α-(Al_*x*_Ga_1−*x*_)_2_O_3_ layers.

## 2. Experimental details

**Fig. 1:**
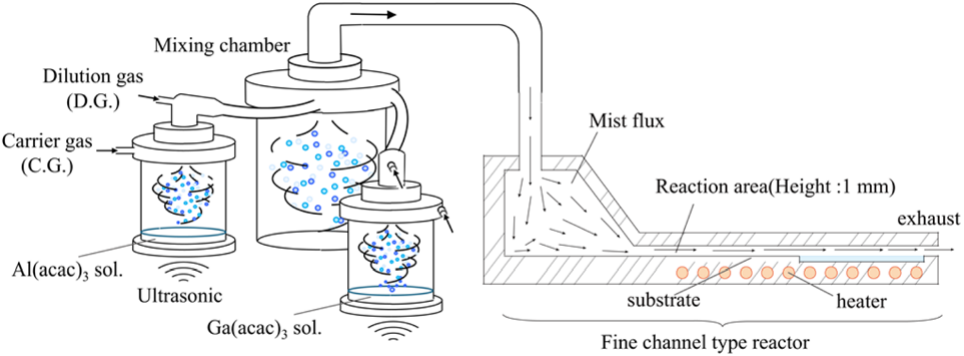
A schematic diagram of the 3G mist CVD system.

To fabricate compositionally step-graded α-(Al_*x*_Ga_1−*x*_)_2_O_3_ layers, it is necessary to determine the growth rate for each α-(Al_*x*_Ga_1−*x*_)_2_O_3_ composition. Therefore, several α-(Al_*x*_Ga_1−*x*_)_2_O_3_ thin films with different compositions were grown and their growth rates were investigated. The growth conditions are summarized in Table 1. Gallium(iii) acetylacetonate (Ga(acac)_3_) and aluminum(iii) acetylacetonate (Al(acac)_3_) were used as precursors for Ga and Al, respectively. They were dissolved in a mixture of deionized water and hydrochloric acid (HCl) at concentrations of 20 mM and 40 mM, respectively. A schematic diagram of the mist CVD system used for α-(Al_*x*_Ga_1−*x*_)_2_O_3_ growth in this work is shown in Fig. 1. Mist generated by the ultrasonic transducers from each solution chamber was well mixed in a dedicated mixing chamber and then supplied to a fine channel reactor. Details of this mist CVD system were described in ref. 38–42. Nitrogen was used as carrier and dilution gases, and α-(Al_*x*_Ga_1−*x*_)_2_O_3_ thin films of different compositions were grown by changing the ratio of Al/Ga carrier gas flow rates. Dilution gas flows were adjusted accordingly to maintain a total flow rate of 6.0 L min^−1^ for each solution chamber, resulting in a total flow rate of 12 L min^−1^ in the reactor. α-(Al_*x*_Ga_1−*x*_)_2_O_3_ thin films were grown on *c*-plane sapphire substrates at 450 °C.

**Table 1:** Growth conditions of α-(Al_*x*_Ga_1−*x*_)_2_O_3_ thin films

Solution	A	B
Solute	Ga(acac)_3_^a^	Al(acac)_3_^b^
Concentration	20 mM	40 mM
Solvent (mixing ratio)	DI water^c^ : HCl^d^ (199 : 1)	
Gas	N_2_	
Substrate	*c*-Plane sapphire	
Growth temperature	450 °C	
Growth system	3G mist CVD	
Ultrasonic transducer	2.4 MHz, 24 V–0.625 A (×3)	

a Gallium acetylacetonate, 99.99%, Sigma-Aldrich.

b Aluminum acetylacetonate, 99.99%, Sigma-Aldrich.

c De-ionized water, Merck Millipore.

d Hydrochloric acid, 35–37%, Wako Pure Chemical Corporation.

## 3. Results and discussion

**Fig. 2:**
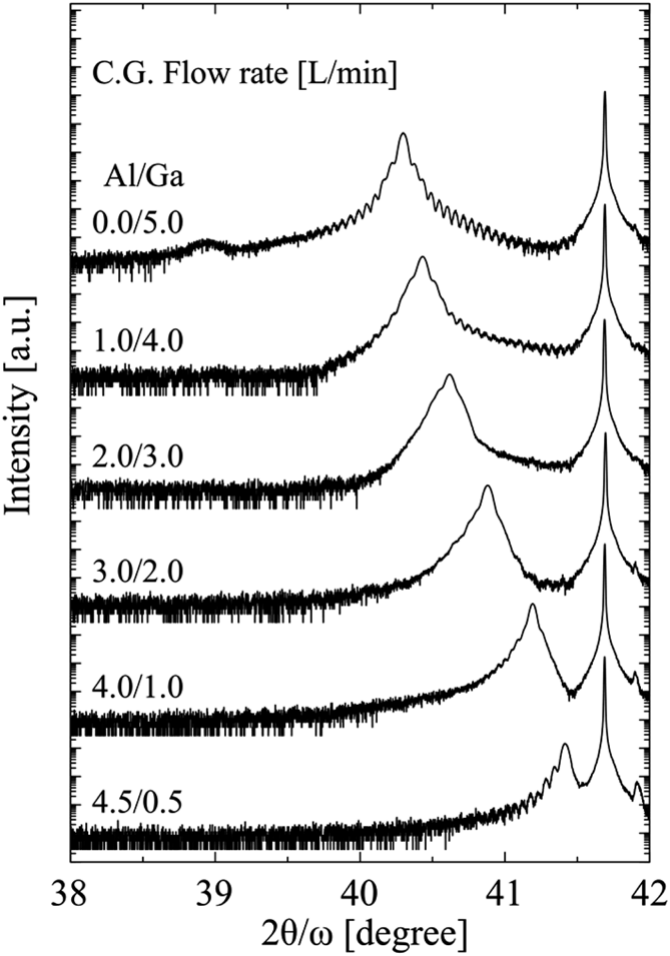
XRD spectra of α-(Al_*x*_Ga_1−*x*_)_2_O_3_ thin films grown by different Al/Ga carrier gas ratio.

**Fig. 3:**
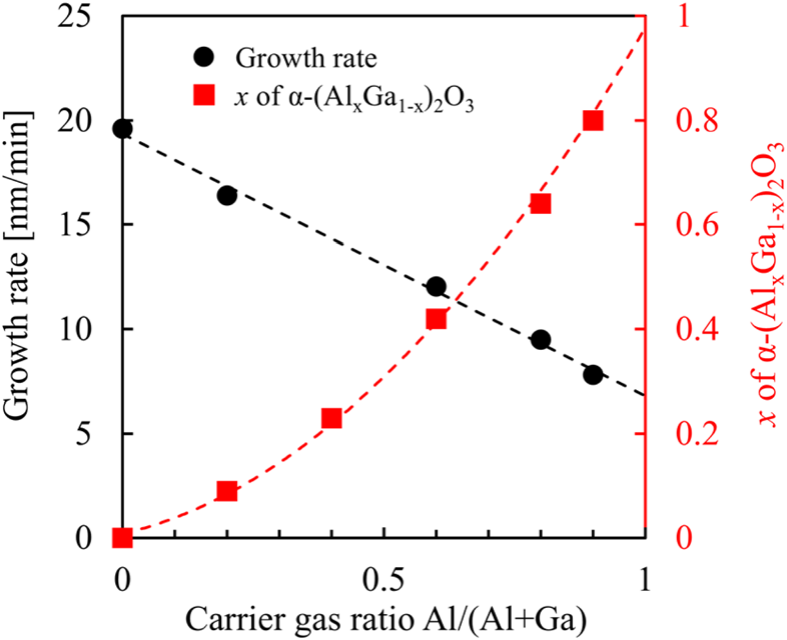
Dependences of growth rates and composition ratios *x* of α-(Al_*x*_Ga_1−*x*_)_2_O_3_ on carrier gas ratio Al/(Al + Ga).

The XRD spectra are shown in Fig. 2. The peaks in the range of 40.3–41.7° correspond α-(Al_*x*_Ga_1−*x*_)_2_O_3_ (0006), and the peak at around 38.9° corresponds to ε-Ga_2_O_3_ (004). The origin of the peak at 41.9° remains unidentified. The peak corresponding to α-(Al_*x*_Ga_1−*x*_)_2_O_3_ (0006) shifted towards the high-angle side as the carrier gas ratio of Al increased, suggesting that the composition of α-(Al_*x*_Ga_1−*x*_)_2_O_3_ could be readily controlled. The α-(Al_*x*_Ga_1−*x*_)_2_O_3_ composition ratio in the film was determined by analyzing the peak positions, as Vegard's law applies to α-(Al_*x*_Ga_1−*x*_)_2_O_3_.^19^ Additionally, the thickness of each α-(Al_*x*_Ga_1−*x*_)_2_O_3_ film was determined by the fringe spacing. The summarized dependencies of composition ratios and growth rates on carrier gas ratios are shown in Fig. 3. Although the thickness of the α-(Al_*x*_Ga_1−*x*_)_2_O_3_ film, which was grown using a carrier gas ratio of Al/Ga = 2.0/3.0 L min^−1^, could not be calculated due to unclear fringes, it was found that the growth rate can be approximated linearly with the Al/Ga carrier gas ratio under the present growth conditions.

**Fig. 4:**
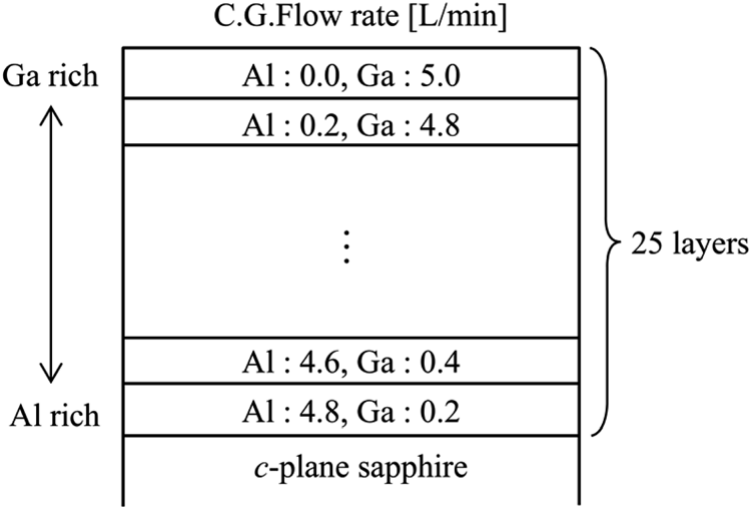
A schematic diagram of the fabricated compositionally step-graded α-(Al_*x*_Ga_1−*x*_)_2_O_3_ layers.

Next, Fig. 4 shows the schematic diagram of the fabricated compositionally step-graded α-(Al_*x*_Ga_1−*x*_)_2_O_3_ layers. The Al carrier gas was decreased by 0.2 L min^−1^ from 4.8 to 0.0 L min^−1^, and the Ga carrier gas was increased by 0.2 L min^−1^ from 0.2 to 5.0 L min^−1^, so that the composition gradually changed from α-Al_2_O_3_ to α-Ga_2_O_3_, forming a total of 25 layers. Table 2 summarizes the carrier gas flow rates and actual film thicknesses of all layers. The thicknesses were equal to or thicker than 50 nm and the growth times were determined based on the results in Fig. 3. α-(Al_*x*_Ga_1−*x*_)_2_O_3_ layers with high Al compositions were thicker than 50 nm due to the presence of compressive strains observed in the high-Al compositions, whereas other layers were 50 nm thick. To grow this structure, the Al and Ga precursor solutions used were the same as those listed in Table 1. Prior to the growth, the *c*-plane sapphire substrate was immersed in a 5 : 2 solution of sulfuric acid and hydrogen peroxide for 10 minutes, followed by a further rinse in deionized water for at least 10 minutes.

**Table 2:** Carrier gas conditions of compositionally step-graded α-(Al_*x*_Ga_1−*x*_)_2_O_3_ layers

Layer	Thickness (nm)	Carrier gas flow rate	
		Al (L min^−1^)	Ga (L min^−1^)
1	80	4.8	0.2
2	75	4.6	0.4
3	70	4.4	0.6
4	65	4.2	0.8
5	60	4.0	1.0
6	58	3.8	1.2
7	56	3.6	1.4
8	54	3.4	1.6
9	52	3.2	1.8
10	50	3.0	2.0

	**50**	**Decrease by 0.2**	**Increase by 0.2**
25	50	0.0	5.0

**Fig. 5:**
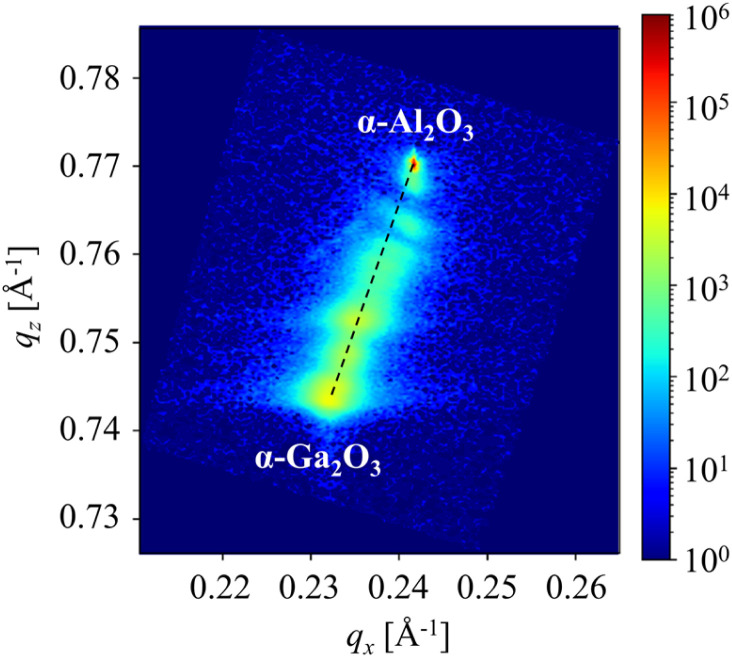
Reciprocal space mapping around (101̄,10) reflections from compositionally step-graded α-(Al_*x*_Ga_1−*x*_)_2_O_3_ layers.

Fig. 5 shows reciprocal space mapping around the (101̄,10) reflection and the dashed line in the figure connects the ideal peak positions of α-Al_2_O_3_ (*q*_*x*_: 0.243, *q*_*z*_: 0.770) and α-Ga_2_O_3_ (*q*_*x*_: 0.232, *q*_*z*_: 0.744).^43^ The peak near the reflection from the sapphire substrate did not follow the dashed line, indicating that high-Al-composition layers seem to have been subjected to a slight in-plane compressive strain. This suggests that a large lattice mismatch occurred between the layer under compressive strain and the relaxed layer. In contrast, in other regions, peaks were observed along the dashed line, indicating that α-(Al_*x*_Ga_1−*x*_)_2_O_3_ layers were fully relaxed.

**Fig. 6:**
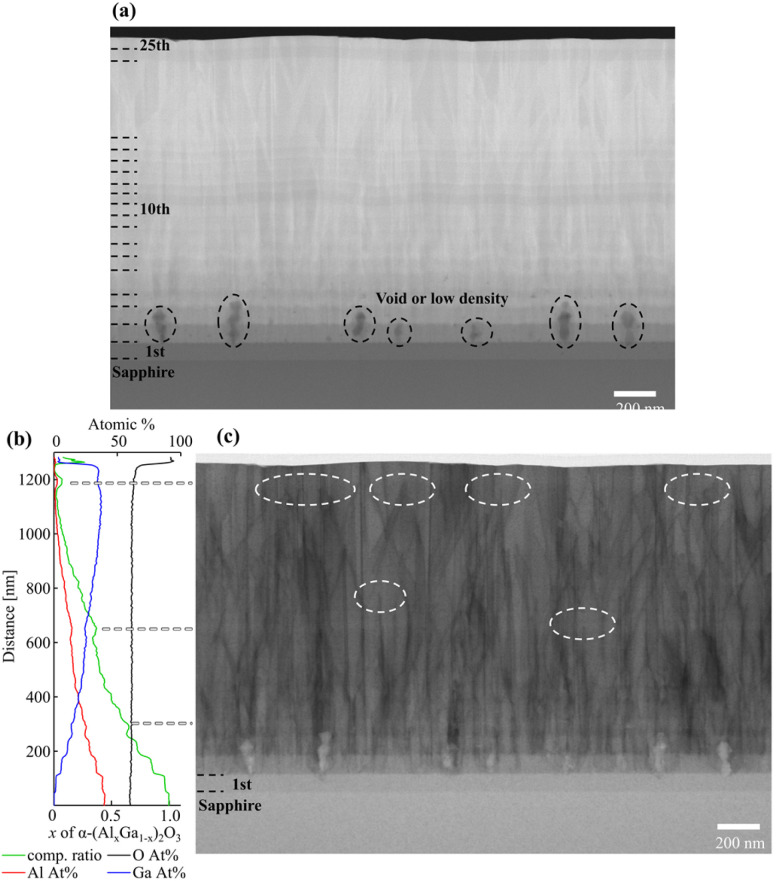
Cross-sectional (a) HAADF-STEM and (c) BF-STEM images, and (b) EDX spectra of compositionally step-graded α-(Al_*x*_Ga_1−*x*_)_2_O_3_ layers.

Fig. 6(a) shows a cross-sectional high-angle annular dark-field scanning transmission electron microscopy (HAADF-STEM) image of the sample thinned to 100 nm thickness. The formation of the 1st to 16th, 24th, and 25th layers could be seen in the order from the sapphire substrate. In addition, voids or low-density regions were observed in the 2nd and 3rd layers. Fig. 6(b) and (c) show energy dispersive X-ray (EDX) and cross-sectional bright-field STEM (BF-STEM) images, respectively. Few dislocations were observed in the 1st layer, but the occurrence of many dislocations was observed starting from the 2nd layer. The dislocation density was estimated to be 7.5 × 10^10^ cm^−2^ in the high-Al-composition region using the Ham method.^44^ The EDX analysis reveals that the first and second layers were α-(Al_0.96_Ga_0.04_)_2_O_3_ and α-(Al_0.84_Ga_0.16_)_2_O_3_, with a lattice mismatch of about 0.57% along the *a*-axis. Although this lattice mismatch value is smaller than that between α-Al_2_O_3_ and α-Ga_2_O_3_ (about 4.60%), the dislocation density is comparable to that of α-Ga_2_O_3_ grown directly on a sapphire substrate.^30^ This indicates that more smoothly compositionally graded layers may be needed or that it might be hard to drastically reduce dislocation density by compositionally step-graded α-(Al_*x*_Ga_1−*x*_)_2_O_3_ layers. On the other hand, it was also found that the dislocations were bent and reduced due to being dislocated out of the TEM sample or pair annihilation in the compositionally graded α-(Al_*x*_Ga_1−*x*_)_2_O_3_. The dislocation density in the high-Ga-composition region was determined to be 3.6 × 10^10^ cm^−2^, which is approximately half of that in the high-Al-composition region, and is similar to the results reported by B. Kim *et al.*^37^ This indicates that the dislocation can be completely bent in the in-plane direction to eliminate the dislocations in the crystal growing on it, or even if not completely bent, the bending can induce pair annihilation, resulting in a reduction in the dislocation density. Based on the EDX results, the *x* of α-(Al_*x*_Ga_1−*x*_)_2_O_3_ decreased gradually from the substrate interface. However, there were three areas where it increased and the dislocation bending, dislocation disappearance from the TEM specimen, or pair annihilation occurred in the vicinity of these areas [Fig. 6(b) and (c)]. This indicates that the dislocation can be bent by compositional changes, suggesting that the use of a superlattice buffer, which alternates compressive and tensile strain by changing the composition of the α-(Al_*x*_Ga_1−*x*_)_2_O_3_, may be effective in reducing the dislocation density.

To elucidate the mechanism of dislocation reduction by strained superlattice buffer layers, structures were fabricated with α-Ga_2_O_3_ inserted in the compositionally step-graded α-(Al_*x*_Ga_1−*x*_)_2_O_3_ buffer layers. Table 3 shows the fabrication conditions. The fabrication conditions for the compositionally step-graded α-(Al_*x*_Ga_1−*x*_)_2_O_3_ layers were identical to those described in Table 2, except for the introduction of four α-Ga_2_O_3_ layers of 50 nm thickness within the aforementioned step-graded α-(Al_*x*_Ga_1−*x*_)_2_O_3_ layers.

**Table 3:** Carrier gas conditions of compositionally step-graded α-(Al_*x*_Ga_1−*x*_)_2_O_3_ layers with insertions of α-Ga_2_O_3_ layers

Layer	Thickness (nm)	Carrier gas flow rate	
		Al (L min^−1^)	Ga (L min^−1^)
1	80	4.8	0.2
2	75	4.6	0.4
3	70	4.4	0.6
4	65	4.2	0.8
5	60	4.0	1.0
6	50	0.0	5.0
7	58	3.8	1.2
8	56	3.6	1.4
9	54	3.4	1.6
10	52	3.2	1.8
11	50	3.0	2.0
12	50	0.0	5.0
13	50	2.8	2.2

	**50**	**Decrease by 0.2**	**Increase by 0.2**
17	50	2.0	3.0
18	50	0.0	5.0
19	50	1.8	3.2
23	50	1.0	4.0
24	50	0.0	5.0
25	50	0.8	4.2
29	500	0.0	5.0

**Fig. 7:**
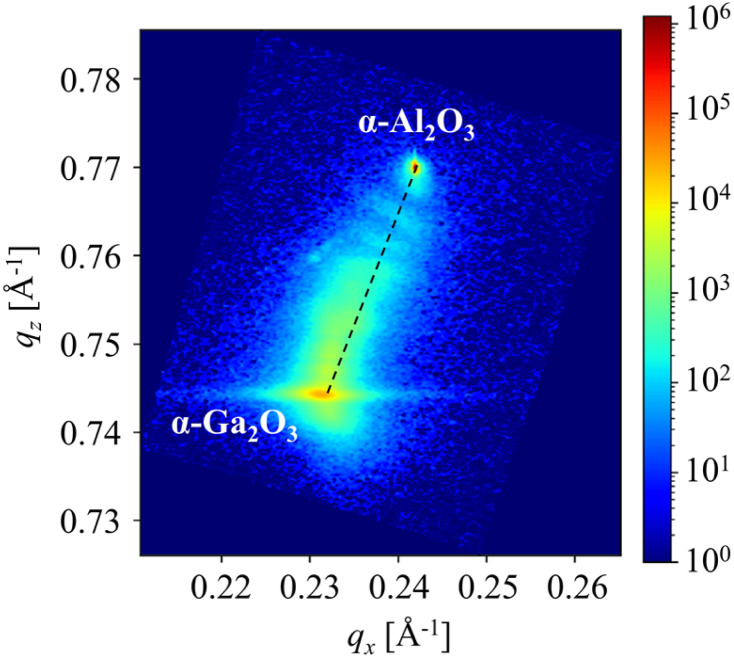
Reciprocal space mapping around (101̄,10) reflections from compositionally step-graded α-(Al_*x*_Ga_1−*x*_)_2_O_3_ layers with insertions of α-Ga_2_O_3_ layers.

Fig. 7 illustrates the reciprocal space mapping around the (101̄,10) reflection of the sample that was fabricated in accordance with the fabrication condition in Table 3. As in Fig. 5, the ideal peak positions of α-Al_2_O_3_ and α-Ga_2_O_3_ are connected by dashed lines. In Fig. 5, the sample exhibited a slight compressive strain in the high-Al-composition region. However, in Fig. 7, it follows the dashed line and was fully relaxed. Conversely, as the Ga composition increased, the α-(Al_*x*_Ga_1−*x*_)_2_O_3_ peak was observed to be situated above and to the left of the dashed line, indicating that it was subjected to tensile strain. Furthermore, the α-Ga_2_O_3_ peak extended to the lower right side, indicating that it was subjected to compressive strain. The insertion of α-Ga_2_O_3_ in the compositionally step-graded α-(Al_*x*_Ga_1−*x*_)_2_O_3_ layers resulted in the complete relaxation of the α-(Al_*x*_Ga_1−*x*_)_2_O_3_ layers in proximity to the substrate interface. However, the remaining α-(Al_*x*_Ga_1−*x*_)_2_O_3_ layers were subjected to tensile strain, and the α-Ga_2_O_3_ layers between the α-(Al_*x*_Ga_1−*x*_)_2_O_3_ layers experienced compressive strain.

**Fig. 8:**
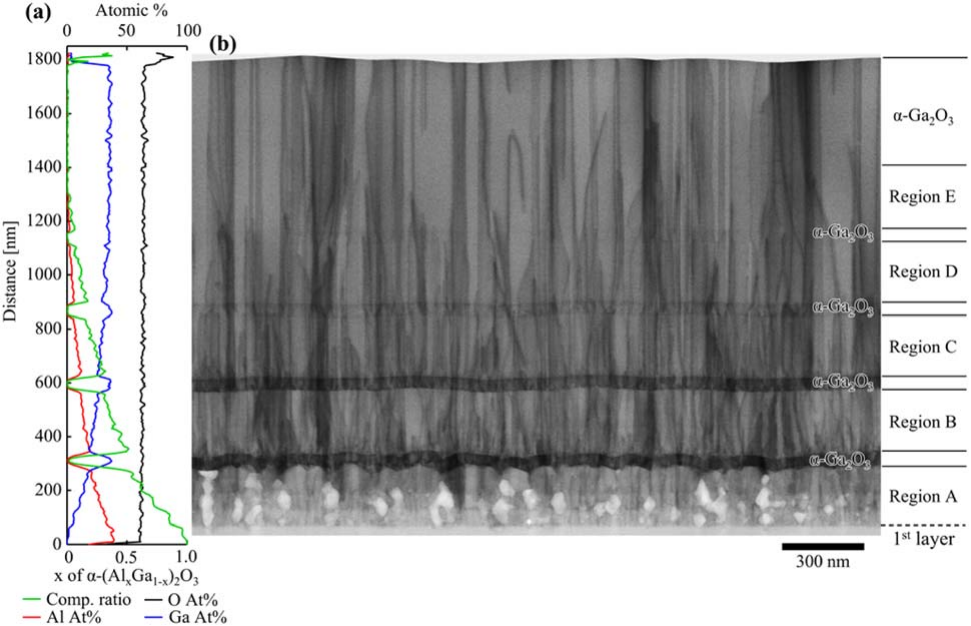
(a) EDX spectra and (b) cross-sectional BF-STEM image of compositionally step-graded α-(Al_*x*_Ga_1−*x*_)_2_O_3_ layers with insertions of α-Ga_2_O_3_ layers.

Fig. 8(a) and (b) show the results of the EDX analysis and the BF-STEM image respectively. As can be seen in Fig. 8(b), the formation of four α-Ga_2_O_3_ layers in the step-graded α-(Al_*x*_Ga_1−*x*_)_2_O_3_ layers were confirmed. Region A revealed numerous dislocations were originated from the second layer and were consistent with the observations shown in Fig. 6(c). These findings suggest that up to α-(Al_0.96_Ga_0.04_)_2_O_3_ can be grown on the *c*-plane sapphire substrate without the presence of dislocations. In contrast, the formation of voids or low-density areas was observed to be more prevalent than in Fig. 6(c). The dislocation densities of regions A, B, C, D, and the surface α-Ga_2_O_3_ were estimated to be 6.56 × 10^10^ cm^−2^, 6.32 × 10^10^ cm^−2^, 5.85 × 10^10^ cm^−2^, 5.21 × 10^10^ cm^−2^, and 3.71 × 10^10^ cm^−2^ respectively. The reduction rates were −3.7% for regions A to B, −7.5% for B to C, −10.8% for C to D, and −28.9% for D to surface α-Ga_2_O_3_ respectively. It is assumed that the smaller compositional alterations were more readily strain without relaxation, resulting in a greater number of dislocations being bent and thus reducing the number of dislocations.

**Fig. 9:**
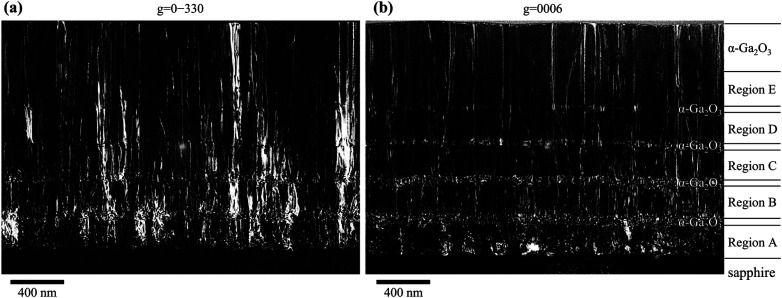
WB-DF images along 〈21̄1̄0〉 zone axis with diffraction vector (a) *g* = 0–330 and (b) *g* = 0006.

Fig. 9(a) and (b) show *g*/3*g* weak-beam dark-field (WB-DF) images acquired along 〈21̄1̄0〉 zone axis with diffraction vectors *g* = 0–330 and *g* = 0006, respectively. In Fig. 9(a), the formation of numerous edge dislocations was confirmed at the interface with the substrate, originating from the second or third layer of step-graded α-(Al_*x*_Ga_1−*x*_)_2_O_3_ layers. These dislocations exhibited a gradual decrease after the α-Ga_2_O_3_ layers grown between α-(Al_*x*_Ga_1−*x*_)_2_O_3_ layers, particularly within regions D and E. In contrast, Fig. 9(b) shows that the formation of screw dislocations originated from the second layer of step graded α-(Al_*x*_Ga_1−*x*_)_2_O_3_ layers. However, a comparison between regions A and B suggests more dislocations in Region B. Given that previous research has indicated that the number of screw dislocations in α-Ga_2_O_3_ grown directly on sapphire substrates was less than 10^7^ cm^−2^,^30^ it can be postulated that the screw dislocations were formed by the insertion of α-Ga_2_O_3_ layers. However, the number of screw dislocations exhibited a decrease in regions C, D, and E, with the α-Ga_2_O_3_ layer serving as the separation point. In particular, a notable reduction in screw dislocations was observed between regions B and C. This indicates that when the thickness of the α-Ga_2_O_3_ layer sandwiched between the α-(Al_*x*_Ga_1−*x*_)_2_O_3_ layers is 50 nm, it is effective to apply a smaller composition change, hence, a smaller strain to reduce edge dislocations and a larger composition change, hence, a larger strain to reduce screw dislocations. Although this study only varied the composition of α-(Al_*x*_Ga_1−*x*_)_2_O_3_, it would be interesting to investigate how dislocations change when the film is strained by varying the thicknesses of each layer, as this also contributes to the strain. From Fig. 9(a) and (b), the edge dislocation density, screw dislocation density, and mixed dislocation density in the surface α-Ga_2_O_3_ layer were estimated to be 1.71 × 10^10^ cm^−2^, 5.20 × 10^9^ cm^−2^, and 1.34 × 10^10^ cm^−2^ respectively. These values were comparable to those obtained in previous studies utilizing quasi-graded α-(Al_*x*_Ga_1−*x*_)_2_O_3_ buffer layers.^36^ The quasi-graded buffer layer was composed of multiple layers of two distinct α-(Al_*x*_Ga_1−*x*_)_2_O_3_ compositions, exhibiting varying thicknesses. In consideration of the findings of the present study, it can be posited that the dislocation density was decreased as a result of the effective strain for dislocation bending in specific layers of a given thickness. It is expected that further reduction of dislocation density will be achieved in future investigations by optimizing the film thickness and the compositional change of α-(Al_*x*_Ga_1−*x*_)_2_O_3_ that applies effective strain to the dislocation bending, as well as by layering multiple layers of these films.

## 4. Conclusions

In conclusion, compositionally step-graded α-(Al_*x*_Ga_1−*x*_)_2_O_3_ layers were grown on a *c*-plane sapphire substrate using mist CVD. Few dislocations were observed in the initial α-(Al_0.96_Ga_0.04_)_2_O_3_ layer; however, numerous dislocations were confirmed from the subsequent α-(Al_0.84_Ga_0.16_)_2_O_3_ layer. The dislocation density was similar to that of α-Ga_2_O_3_ grown directly on sapphire substrates. However, the dislocation density in the high-Ga-composition α-(Al_*x*_Ga_1−*x*_)_2_O_3_ layer was decreased by approximately 50% due to the dislocation bending in the α-(Al_*x*_Ga_1−*x*_)_2_O_3_ layers. This dislocation bending was observed in the α-(Al_*x*_Ga_1−*x*_)_2_O_3_ layers where alternating compressive and tensile stresses were applied as a result of composition differences. To clarify the mechanism of dislocation bending, compositionally step-graded α-(Al_*x*_Ga_1−*x*_)_2_O_3_ layers inserted with several α-Ga_2_O_3_ layers was fabricated. The dislocations were reduced following the insertion of α-Ga_2_O_3_ layers into the compositionally step-graded α-(Al_*x*_Ga_1−*x*_)_2_O_3_. The results of the reciprocal space mapping indicate that this α-Ga_2_O_3_ layer was strained, and it is proposed that the dislocations are bent by the strain caused by the compositional change of α-(Al_*x*_Ga_1−*x*_)_2_O_3_, resulting in the reduction of dislocations. The differentiation of dislocation types according to the WB-DF images revealed that smaller strains resulting from smaller compositional changes were effective in reducing edge dislocations, whereas larger strains resulting from larger compositional changes were effective in reducing screw dislocations. It is expected that future investigations will achieve a further reduction in dislocation density by optimizing the film thickness and the compositional change of α-(Al_*x*_Ga_1−*x*_)_2_O_3_, which applies effective strain to the dislocation bending. Additionally, the use of multiple layers of these films will be examined as a means of further reducing dislocation density.

## Data availability

The data supporting this article is included in the manuscript.

## Author contributions

Tatsuya Yasuoka – conceptualization, methodology, investigation, visualization, and writing original draft; Hiromu Susami – investigation; Li Liu – methodology and investigation; Giang T. Dang – methodology and writing review & editing; Toshiyuki Kawaharamura – conceptualization, project administration, supervision, methodology, and writing review & editing.

## Conflicts of interest

There are no conflicts to declare.
